# Dynamics Rationalize Proteolytic Susceptibility of the Major Birch Pollen Allergen Bet v 1

**DOI:** 10.3389/fmolb.2020.00018

**Published:** 2020-02-20

**Authors:** Anna S. Kamenik, Florian Hofer, Philip H. Handle, Klaus R. Liedl

**Affiliations:** Center for Molecular Biosciences Innsbruck, Institute of General, Inorganic and Theoretical Chemistry, University of Innsbruck, Innsbruck, Austria

**Keywords:** allergen proteins, molecular dynamics simulations, unfolding, proteolytic cleavage, enhanced sampling, Markov state models

## Abstract

Proteolytic susceptibility during endolysosomal degradation is decisive for allergic sensitization. In the major birch pollen allergen Bet v 1 most protease cleavage sites are located within its secondary structure elements, which are inherently inaccessible to proteases. The allergen thus must unfold locally, exposing the cleavage sites to become susceptible to proteolysis. Hence, allergen cleavage rates are presumed to be linked to their fold stability, i.e., unfolding probability. Yet, these locally unfolded structures have neither been captured in experiment nor simulation due to limitations in resolution and sampling time, respectively. Here, we perform classic and enhanced molecular dynamics (MD) simulations to quantify fold dynamics on extended timescales of *Bet v 1a* and two variants with higher and lower cleavage rates. Already at the nanosecond-timescale we observe a significantly higher flexibility for the destabilized variant compared to *Bet v 1a* and the proteolytically stabilized mutant. Estimating the thermodynamics and kinetics of local unfolding around an initial cleavage site, we find that the Bet v 1 variant with the highest cleavage rate also shows the highest probability for local unfolding. For the stabilized mutant on the other hand we only find minimal unfolding probability. These results strengthen the link between the conformational dynamics of allergen proteins and their stability during endolysosomal degradation. The presented approach further allows atomistic insights in the conformational ensemble of allergen proteins and provides probability estimates below experimental detection limits.

## Introduction

With more than 30% of the population affected, immunoglobulin-E-mediated allergy is the most frequent immune disorder worldwide (Valenta, 2002; Valenta et al., 2010; Curin et al., 2018). Still, the determinants of what makes a protein an allergen are largely unknown (Scheurer et al., 2015; Verhoeckx et al., 2019). It has been demonstrated repeatedly that proteins with substantial similarity in sequence and structure are able to trigger an entirely different immune response (Mitropoulou et al., 2018; Eichhorn et al., 2019; Seutter von Loetzen et al., 2019; Tscheppe et al., 2020). Thus, the allergenic potential of a protein often cannot be rationalized, nor predicted, based on sequence or structural similarity to known allergens (Lu et al., 2018). However, one decisive feature biasing the immunologic response to a protein antigen is its proteolytic susceptibility (Toda et al., 2011; Apostolovic et al., 2016; Machado et al., 2016; Scheiblhofer et al., 2017; Wolf et al., 2018). Hence, reliable experimental and computational models of proteolytic susceptibility in the endosome represent promising tools to understand, and possibly even predict, what makes a protein an allergen.

In a study by Machado et al. (2016) point mutations were introduced to the major birch pollen allergen Bet v 1, which increased its proteolytic stability and also entirely modulated the resulting immune response. This trend was explained in the context of allergen processing and presentation (Freier et al., 2015; Machado et al., 2016; Scheiblhofer et al., 2017). Exogenous proteins enter antigen presenting cells, such as dendritic cells, via endocytosis. In the endosome, the protein is then cleaved into small peptide fragments by endolysosomal proteases, such as cathepsin S (Watts, 2001; Hsing and Rudensky, 2005; Conus and Simon, 2010; Egger et al., 2011; Freier et al., 2015). These fragments are then loaded onto major histocompatibility complex molecules type 2 (MHC2) and transported to the cell surface. The recognition of this MHC2-peptide complex by T-helper cells then determines the subsequent immune response (Watts, 2004; Valenta et al., 2010). Thus, proteolytic cleavage of the exogenous antigen is a crucial step in this mechanism and is discussed as the origin of the varying allergenic potential of the studied Bet v 1 variants (Thai et al., 2004; Machado et al., 2016; Wolf et al., 2018). Only antigens with an “optimal” stability are able to induce an allergic response on T-cell level. It has been postulated that proteins of low proteolytic stability are already cleaved in the early endosome, leading to a protective immune response. High proteolytic stability, on the other hand, leads to late degradation in the lysosome and thus fails to induce the required T cell polarization (Scheiblhofer et al., 2017). Consequently, targeted design of Bet v 1 variants with specific proteolytic susceptibilities is a promising approach in allergen-specific immunotherapy (Curin et al., 2018).

Interestingly, none of the mutations introduced by Machado et al. (2016) were located in the initial cleavage sites. Hence, the recognition pattern of the protease remains unchanged. Still, the cleavage rates already differ substantially after introduction of a single point mutation, i.e., the exchange of aspartate 69 with isoleucine (D69I). Additionally, the initial cleavage sites are mostly located within stable secondary structure elements, e.g., the main initial cleavage site of Bet v 1 is found in the first alpha helix between residues A16 and I23 (Egger et al., 2011; Freier et al., 2015). Yet, for the involved proteases it is sterically impossible to bind the allergen in this conformation (Madala et al., 2010). Thus, the allergen inevitably has to unfold locally to become susceptible to proteolysis and lead to the observed cleavage pattern.

It is well-established that proteins in solution fluctuate between diverse conformational states of varying probability, including (partially) unfolded states (Karplus and Weaver, 1976; Henzler-Wildman and Kern, 2007; Boehr et al., 2009; Dror et al., 2012; Latorraca et al., 2017). We thus surmise that the equilibrium between folded and partially unfolded conformational states of a protein, is linked to its proteolytic susceptibility. In line with this concept, increased cleavage rates indicate a shift of the ensemble toward partially unfolded conformations, whereas a stabilizing mutation decreases the probability of local unfolding (Freier et al., 2015). Substantial efforts have led to a multitude of structural models of allergen proteins with a Bet v 1-like fold from X-ray crystallography as well as NMR structure refinements (Gajhede et al., 1996; Schweimer et al., 1999; Aalberse, 2000; Ahammer et al., 2017; Moraes et al., 2018; Jacob et al., 2019). However, despite the utmost importance of local unfolding for proteolytic cleavage, no structural models depicting partially unfolded conformations have yet been solved. This might indicate a low probability of the partially unfolded state within the solution ensemble, below the detection limit of X-ray crystallography and NMR structure refinements (Hart et al., 2016). However, using dynamic NMR experiments, including backbone amide ^15^N relaxation dispersion and hydrogen-deuterium exchange experiments, in combination with MD simulations we were able to capture a relation between conformational dynamics and varying cleavage rates of Bet v 1 variants (Grutsch et al., 2017). While, increased flexibility was observed around initial cleavage sites, the obtained data did not allow for a structural model of a partially unfolded state.

As described above, Bet v 1 is among the most extensively studied allergen proteins in experiment, computational models and clinical studies (Spangfort et al., 2003; Bollen et al., 2010; Berkner et al., 2014; Garrido-Arandia et al., 2014; Grutsch et al., 2014; Kitzmuller et al., 2015; Kamenik et al., 2016; Moingeon et al., 2016; Biedermann et al., 2019). With an estimated 100 million of individuals suffering from birch pollen allergy, it is also one of the clinically most relevant allergens (Hartl et al., 2004). Most intriguingly for the present study, the degradation patterns of Bet v 1 and a multitude of its mutants have been studied with a degradome assay (Egger et al., 2011; Machado et al., 2016). This assay was specifically designed to mimic endolysosomal degradation of allergen proteins and allows for a time-resolved quantification of proteolytic susceptibility. Thereby, detailed information on location and quantity of the initial scissile sites became accessible (Egger et al., 2011). Several studies have been published focusing on the proteolytic susceptibility and fold stability of Bet v 1 variants (Pree et al., 2007; Husslik et al., 2015; Apostolovic et al., 2016; Ferrari et al., 2016; Machado et al., 2016; Pekar et al., 2018). In addition to variations in sequence also environmental factors, such as nitration or ligand binding, have been shown to impact proteolytic susceptibility as well as the immunogenicity of Bet v 1 and allergen proteins in general (Mogensen et al., 2002; Ackaert et al., 2014; Asam et al., 2014; Grutsch et al., 2014; von Loetzen et al., 2014, 2015; Foo et al., 2019; Soh et al., 2019).

Here, we analyze the fold dynamics of the Bet v 1.0101 (*Bet v 1a*) wild-type protein and two variants with varying stability in the context of proteolytic susceptibility. We study the isoform Bet v 1.0102 (*Bet v 1d*) as destabilized variant. Differing in seven amino acids, *Bet v 1d* shares a sequence identity of 95.6% with the wild-type protein *Bet v 1a* (Wagner et al., 2008). Only one mutation is found in the vicinity of the major initial cleavage site (F30V). However, this exchange cannot explain the considerable difference found for the cleavage rate at this site (Egger et al., 2011; Freier et al., 2015). Even more fascinating is the above-mentioned point-mutant *Bet v 1a-D69I*. This mutation is located far from the cleavage site, both in sequence and in structure. Nevertheless, the decrease of the degradation rate compared to the wild-type protein is substantial (Machado et al., 2016). In an effort to quantify distinctions in structural fluctuations within the native state ensemble we perform classic molecular dynamics (MD) simulations. We thereby compare the stability of the three Bet v 1 variants (*Bet v 1a, Bet v 1d*, and *Bet v 1a-D69I*) based on dynamics observed at the nano- to microsecond timescale. We characterize the dynamics of the native state ensemble at pH 5, mimicking the acidic conditions in the endolysosomal antigen-processing compartment, with classic MD (cMD) simulations. In a previous study on Bet v 1 variants described above, we have applied a similar workflow, combined with NMR relaxation experiments to study a broach range of dynamics. With the use of NMR relaxation dispersion measurements, we found the initial cleavage site in *Bet v 1 d* to be more dynamic than in *Bet v 1a*, particularly on the millisecond timescale. Yet, all previous calculations were adjusted to the experimental conditions and thus protonation states resembling pH 7 were applied (Machado et al., 2016; Grutsch et al., 2017). In this study however, we aim at modeling ensembles of Bet v 1 variants, as they are expected to occur at pH 5. This decrease in pH substantially alters the protonation states, and thus H-bond networks as well as coulomb interactions within each structure (Hofer et al., 2019). Hence, the presented results are not directly comparable to previously published findings. Additionally, we present the first approach to capture the partial unfolding in allergen proteins and estimate the associated thermodynamics and kinetics. It is expected that the conformational rearrangements within the entire fold, which are inherently linked to the partial unfolding of the initial cleavage site, are extensive. Furthermore, as described above, we expect that the probability of the partially unfolded state of the studied Bet v 1 variants is low compared to the folded state at the applied conditions (37°C, 1 bar, pH 5). Thus, it is highly unlikely to observe this process on timescales accessible to classic MD simulations. Hence, we employ an enhanced sampling technique, well-tempered metadynamics MD (MDMD) (Barducci et al., 2008; Laio and Gervasio, 2008; Leone et al., 2010), to drive the transition from a folded to a locally unfolded conformational state.

The applied workflows allow us on the one hand to study motions and interactions within the folded state, where barriers are low and dynamics on the ns- to μs-timescale can be monitored (Bowman, 2016). On the other hand, we also profile the slow motions resulting in partial unfolding of the Bet v 1 variants in atomistic detail. By calculating thermodynamic and kinetic properties from both approaches we rationalize the experimentally determined differences in proteolytic susceptibility.

## Methods

### Simulation Setup

The starting structures for the wild-type protein, *Bet v 1a*, and its isoform *Bet v 1d* were prepared from the respective available crystal structures [PDB codes 4A88 (Kofler et al., 2012) and 1FM4, respectively (Marković-Housley et al., 2003)] with the program MOE (Molecular Operating Environment) (Labute, 2009; MOE, 2019). All ions, crystallization agents and water molecules were removed. The D69I point mutant was created with the program MOE via single point mutation, followed by a short backbone relaxation, since no experimental structures were available.

The LEaP module of the AmberTools19 program suite (Case et al., 2018) was used to add missing hydrogens and create topology and coordinate files. The AMBER ff14SB force field (Maier et al., 2015) was used for all simulations. The protein was solvated in TIP3P water (Jorgensen et al., 1983) with a cubic box maintaining a minimum wall distance of 10 Å. Prior to production, all systems were equilibrated according to an elaborate protocol previously developed in our group (Wallnoefer et al., 2010).

All simulations were performed with the GPU implementation of the pmemd module of AMBER 18 (Salomon-Ferrer et al., 2013). To keep the target temperature of 310 K, the Langevin thermostat (Adelman and Doll, 1976) was used with a collision frequency of 2 ps^−1^. Furthermore, we used the Berendsen barostat with a relaxation time of 2 ps to maintain atmospheric pressure (Berendsen et al., 1984). The SHAKE algorithm was used to constrain all bonds involving hydrogens and allow the use of a 2 fs time step (Ryckaert et al., 1977) for the simulation. Long range electrostatics were treated with the particle-mesh Ewald method (PME) and a non-bonded cutoff of 8 Å was used (Darden et al., 1993).

We used well-tempered metadynamics MD (MDMD) (Barducci et al., 2008) as implemented in GROMACS version 2016.4 (Abraham et al., 2015), i.e., plumed 2.4.0 (Tribello et al., 2014), to achieve a transition from the folded to partially unfolded states. The underlying idea of MDMD is to fill up potential energy minima with a time-dependent Gaussian bias potential, which consequently lowers the energetic barriers along a selected reaction coordinate. Thereby, the conformational space of a protein is sampled much more efficiently and slow motions become accessible (Laio and Parrinello, 2006; Laio and Gervasio, 2008). In well-tempered MDMD additionally the height of the Gaussians is adaptively changed (Barducci et al., 2008). After extensive testing, the distance between the Cαs of the residues flanking the initial cleavage site, i.e., A16 and I23, was identified as suitable reaction coordinate resulting in the most efficient sampling of the local unfolding process. Gaussian functions were deposited every 1,000 steps with a height of 10.0 kcal/mol, a width of 0.35 and biasfactor of 30. To capture a diverse set of unfolding pathways we performed ten independent 10 ns well-tempered MDMD simulations for each system. For each run we assigned new starting velocities (see Supplementary Figure 1A).

The introduction of a bias potential renders the calculation of accurate thermodynamics and especially kinetic properties rather challenging (Stelzl et al., 2018). We evaluated the energetics of the captured unfolding pathways by reweighting the reaction coordinate as described by Tiwary and Parrinello (2015). However, as depicted in Supplementary Figure 2, the individual free energy curves of each system vary substantially. We surmise that this behavior stems from the varying unfolding pathways captured in the individual simulations. Due to the large differences between the runs for each system we were not able to perform reliable comparisons between systems. While the straight-forward approach of boosting the end-to-end distance of the cleavage site led to a highly efficient sampling of the unfolding process, this reaction coordinate fails to reflect this complex process in terms of reweighted free energies. We circumvent these limitations by seeding a multitude of cMD simulations along the captured transition pathway. To do so, we clustered the captured ensembles based on Cα positions using an average-linkage hierarchical clustering algorithm with a distance cutoff of 4 Å. The resulting representative structure of each cluster was then used as a starting structure for a cMD simulation of 500 ns length applying the same setup as described above (see Supplementary Figure 1B). We then combined the cMD simulations of each Bet v 1 variant by constructing a Markov state model (MSM) (Prinz et al., 2011). The construction of the MSM allows to quantify thermodynamic and kinetic properties of each ensemble without the intrinsic bias resulting from the seeding process (Bowman et al., 2013; Kohlhoff et al., 2014). Similar workflows have already been proven to be extremely efficient and highly reliable (Noe et al., 2009; Nedialkova et al., 2014; Biswas et al., 2018; Fernandez-Quintero et al., 2018; Sun et al., 2018; Zimmerman et al., 2018).

### Analysis

The programs cpptraj and pytraj from the AmberTools19 suite, combined with in-house python scripts were used for analysis. The number of native contacts was calculated using mdtraj as described by Best et al. (2013). Here, we used each N and O in the backbone with a distance below 3 Å to define a native contact. We performed principle component analysis (PCA) based the distances between backbone N and O in helix 1 and helix 2 to compare structural variances. For the construction of the PCA space we combined the trajectories from all Bet v 1 variants and then projected the individual structural data of each variant onto the two largest eigenvectors (PC1 and PC2). PCA identifies the motions which account for the largest structural variance. Time-lagged independent component analysis (tICA), on the other hand, is a dimensionality-reduction technique which detects the slowest-relaxing degrees of freedom (Molgedey and Schuster, 1994; Chodera and Noe, 2014; Schwantes et al., 2016). The tICA space thus facilitates a kinetic clustering, which is prerequisite for building MSMs (Bowman et al., 2013; Shukla et al., 2015). We employed the same input features for tICA as described for PCA. We applied the implemented k-means clustering algorithm to discretize the tICA space into 100 microstates. A lag time of 10 ns was chosen, since the estimated slowest timescales are approximately independent of the lag time at that point (Pande et al., 2010; Bowman et al., 2013) (see Supplementary Figure 2). Subsequently Perron cluster cluster analysis (PCCA+) was performed to achieve an intuitive coarse-grained representation of the Bayesian MSM (Röblitz and Weber, 2013). To estimate the reliability of the calculated models we performed typical MSM validations, i.e., plotting the implied timescales as a function of lag-time as well as a Chapman-Kolmogorov test (Supplementary Figure 3). Furthermore, we show uncertainty estimates for the presented models (Supplementary Tables 1, 2) as well as the state probabilities as a function of lag time (Supplementary Figure 5). PCA, tICA and MSM analyses as well as plotting of the respective results were achieved with the pyEMMA package version 2.5.6 (Scherer et al., 2015).

## Results

### μs-Dynamics in the Folded State

**Figure 1:**
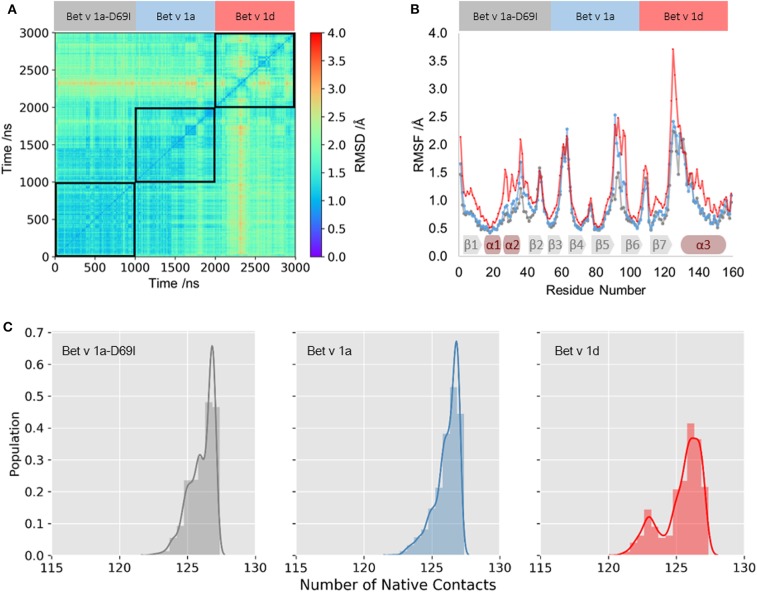
Fold dynamics of Bet v 1 variants. **(A)** The conformational space captured in 1 μs cMD simulations of the stabilized variant *Bet v 1a-D69I*, wild-type protein *Bet v 1a* and the destabilized variant *Bet v 1d*, respectively, was profiled via a 2D-RMSD analysis based on all Cα atoms. **(B)** To quantify the local differences in flexibilities we calculate the RMSF for each residue based all Cα atoms. The secondary structure elements found in the crystal structure are depicted on the bottom. **(C)** From the distribution of native contacts between backbone N and O atoms of helix1 and helix2 we calculate entropies as a measure of backbone rearrangements.

We profile differences in the dynamics within the fully folded state of the three Bet v 1 variants with classic MD (cMD) simulations of 1 μs length. In Figure 1A we visualize similarities in the captured conformational ensembles with a 2D-RMSD plot; Blue indicates high structural similarity to the crystal structure and red denotes structural rearrangements. This analysis depicts only small fluctuations in the structure of the wild-type protein *Bet v 1a*. The stabilized mutant *Bet v 1a-D69I*, shows even subtler fold dynamics, which largely concur with the wild-type ensemble. In contrast, the 2D-RMSD of *Bet v 1d*, the destabilized variant, points toward a larger conformational diversity within the captured ensemble. To quantify the structural fluctuations observed in the simulations, we calculate the RMSF for each system. We find comparable dynamics for the wild-type protein and the stabilized mutant, only between residue 90 and 100 the wild-type protein is slightly more mobile. *Bet v 1d* on the other hand shows an overall higher flexibility than the other variants. This trend is also reflected by the number of native contacts formed during the simulations (see Figure 1C). Clearly, for the less stable variant, *Bet v 1d*, the distribution of native contacts is broader and shifted toward less contacts. Furthermore, we calculate the Shannon entropy of the distributions in this contact space as an alternate measure of flexibility. With an entropy of 17.4 J/ (mol·K) *Bet v 1d* again arises as the most flexible system, compared to *Bet v 1a* with 15.1 J/ (mol·K) and *Bet v 1a-D69I* with 13.8 J/ (mol·K).

**Figure 2:**
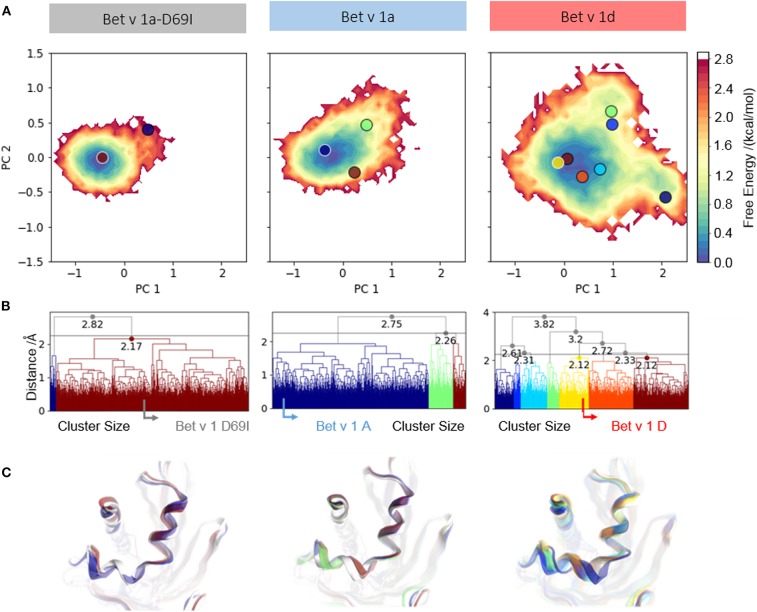
Conformational space of residues around the early cleavage site of Bet v 1 variants. **(A)** The captured ensembles were color-coded according to Gibbs free energies and projected onto the first two eigenvectors of the combined PCA space. **(B)** Clustering of the conformational space of helix 1 and helix 2 resulted in two clusters for the stabilized variant *Bet v 1a-D69I*, three clusters for the wild-type protein *Bet v 1a* and seven clusters for the destabilized variant *Bet v 1d*, at a distance cut-off of 2.5 Å. The native structure of each variant is denoted with an arrow in the respective dendrogram. **(C)** Representative structures were extracted from each cluster to visualize conformational differences and were projected onto the PCA space.

To further analyze the captured conformational space of each system, focusing on the early cleavage site, we construct a free energy surface in a combined PCA space as described in the methods section (Figure 2A). We find one narrow minimum, which represents the conformational ensemble of *Bet v 1a-D69I*. In comparison to this, the conformational space of *Bet v 1a* is slightly broader. However, the observed dynamics are still represented as one broad minimum in the combined PCA space. The ensemble of *Bet v 1d* on the other hand, is by far the most diverse and shows transitions between three separated minima.

We cluster each trajectory applying the same distance cutoff of 2.5 Å to gain further insight into the captured structural rearrangements (Figures 2B,C). The increase in the number of resulting clusters from *Bet v 1a-D69I* over *Bet v 1a* to *Bet v 1d* indicates more diversity in the respective underlying data. As depicted in Figure 2C, the resulting representative structures show only marginal deviation for *Bet v 1a-D69I* and *Bet v 1a*. For *Bet v 1d* we find a slight shift of both helices. However, the conformational changes observed in 1 μs of cMD simulation are small, also for *Bet v 1d*, the proteolytically least stable system. Additionally, we project the representative structures from each cluster onto the combined PCA space in Figure 2A to link structural and energetic information.

### Local Unfolding Dynamics

**Figure 3:**
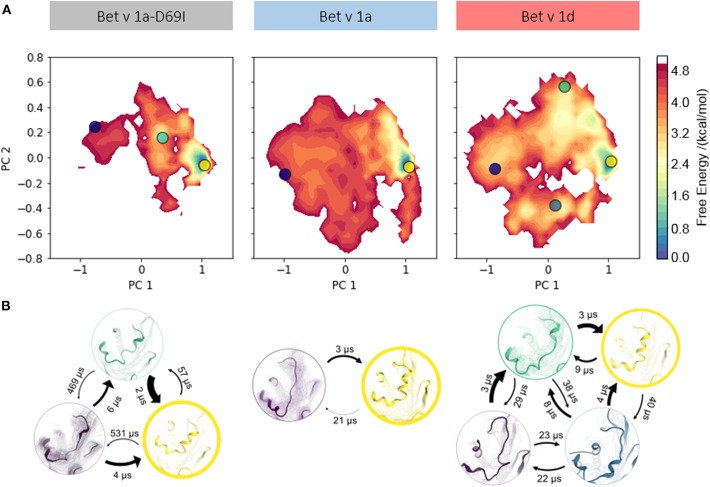
**(A)** Free energy landscape during local unfolding. The accumulated structural information from the seeded cMD simulations were color-coded according to the reweighted free energies and projected onto the first two eigenvectors of the combined PCA space. **(B)** We constructed an MSM of the partial unfolding process of each variant. Estimating the state populations of the stabilized variant *Bet v 1a-D69I*, the wild-type protein *Bet v 1a* and the destabilized variant *Bet v 1d*, we found 95 ± 2, 87 ± 3, and 65 ± 6% of the ensembles in the folded state, respectively.

To profile whether we can capture a relation between proteolytic susceptibility and the thermodynamics and kinetics of partial unfolding events, we apply a combination of enhanced sampling techniques and classic MD simulations (see section Methods). This approach allows us to profile distinct features along the unfolding pathway for each Bet v 1 variant. To compare the structural diversities of each ensemble we again perform PCA on the combined trajectories. In Figure 3A structural data from each variant is projected onto the two largest eigenvectors and color-coded according to the MSM reweighted free energy. Also on this extended timescale the stabilized mutant *Bet v 1a-D69I* clearly shows a narrower free energy surface than the wild-type protein *Bet v 1a*. The conformational landscape of *Bet v 1a-D69I* shown in the PCA space displays one deep and distinct free energy minimum at (1, 0) and two significantly less favorable minima around (0.5, 0.2) and (−1, 0.2). The free energy landscape of the residues around the early cleavage site of *Bet v 1a* is much broader than for *Bet v 1a-D69I*, indicating a higher structural diversity. However, also for *Bet v 1a* we only observe one deep and distinct minimum in free energy around (1, 0). The remaining surface is characterized by rather flat and unfavorable areas. The conformational space sampled by *Bet v 1d* is only slightly broader than the wild-type, with the most distinct minimum again around (1, 0). However, in terms of free energy we evidently find an overall shallower and much more favorable surface for *Bet v 1d*. On the one hand, this analysis suggests a higher probability of partially unfolded states within the *Bet v 1d* ensemble. On the other hand, the projection onto the PCA space also implies lower barriers between the individual minima, i.e., conformational states. These observations are in line with the transition timescales estimated from the MSM (Figure 3B). While for *Bet v 1a* and *Bet v 1a-D69I* the fastest unfolding process takes place at timescales around 21 μs and 57 μs, *Bet v 1d* unfolds already within 9 μs. To obtain a more detailed view on the partial unfolding mechanism we additionally analyze the most probable transition pathway using the net reactive flux (see Supplementary Figure 2). Furthermore, we retrieve the probabilities of each conformational state via the MSM stationary distribution of each model. For the folded state a probability of 95 ± 2% is estimated for the stabilized mutant *Bet v 1a-D69I*, 87 ± 3% for *Bet v 1a*, and 65 ±6 % for *Bet v 1d*.

## Discussion

One feature which has been shown to play a decisive role in the immunogenicity of birch pollen allergens is proteolytic stability (Scheiblhofer et al., 2017). However, it is still challenging to rationalize a protein's resilience or susceptibility to proteolytic degradation based on its sequence or a single structure alone. Here, we present a simulation-based approach to illustrate and quantify features along the transition path to a partially unfolded state, which is imperative for proteolytic cleavage.

As illustrated in Figure 1, we apply various metrics to quantify the structural fluctuations of Bet v 1 variants within the folded state. The results from 2D-RMSD and RMSF clearly identify the ensemble of the proteolytically least stable variant *Bet v 1d* as structurally most diverse. On the other hand, a difference in the dynamics of *Bet v 1a* and the stabilized mutant *Bet v 1a-D69I* is only marginally visible from these analyses. While the 2D-RMSD illustrates varying structural rearrangements of the overall fold, the RMSF allows to localize differences of flexibilities within each structure. One of the first cleavage sites which was experimentally observed is located in the vicinity of helix 1 and helix 2, between the residues A16 and I23 (Egger et al., 2011; Freier et al., 2015; Machado et al., 2016). Strikingly, this region shows significantly increased local flexibilities for *Bet v 1d* in the RMSF analysis (Figure 1B). We thus, concentrate our analysis on the dynamics observed in close vicinity to this early cleavage site.

To profile structural deviations from the crystal structure of Bet v 1 we determine the number of native contacts formed and lost during the simulations for helix 1 and helix 2 (Best et al., 2013). This alignment-independent metric intuitively characterizes the fold stability of a given protein and can also be used for an in-depth study of individual secondary structure elements. As shown in Figure 1C, we observe a significantly broader distribution of formed native contacts in helix 1 and 2 for *Bet v 1d*. For the wild-type protein *Bet v 1a* and the stabilized variant *Bet v 1a-D69I* the number of native contacts show similar trends and are clearly shifted to higher values. These observations again suggest *Bet v 1d* as the most flexible variant. To quantify the distinctions between these distributions, we calculate the Shannon entropy. The increase in entropy from *Bet v 1a-D69I*, over *Bet v 1a* to *Bet v 1d* implies that the proteolytically least stable variant, *Bet v 1d*, is also the least stable in terms of native contacts.

Additionally, we compare structural variances and the associated energetics for each ensemble by constructing free energy surfaces in the combined PCA space (Figure 2A). The captured conformational space of *Bet v 1d* is separated into three distinct minima and significantly more extensive compared to the Bet v 1 variants with lower cleavage rates. *Bet v 1a* and *Bet v 1a-D69I* show very similar free energy landscapes, with only one deep minimum in free energy and *Bet v 1a* covering a slightly broader space in this projection. The structural clustering with the same cutoff distance for all systems and the subsequent projection of the representative structures onto the combined PCA space allows for a structural interpretation of the trends discussed above. The increasing number of clusters from *Bet v 1a-D69I*, over *Bet v 1a*, to *Bet v 1d*, and their respective populations again indicate an increase of structural fluctuations within the early cleavage site. However, upon visual inspection of the representative structures it becomes apparent that the involved structural rearrangements are almost negligible. Only for *Bet v 1d* we find a notable shift in the orientation of helix 2. Nevertheless, all of the applied metrics to characterize the dynamics of the three Bet v 1 variants, clearly identify the structural ensemble of *Bet v 1d* as most diverse. This result indicates that already dynamics on the ns- to μs-timescale could be decisive for exceptionally high proteolytic cleavage rates.

Nevertheless, the main scope of this study is to characterize the partial unfolding process, which is prerequisite for proteolytic cleavage of Bet v 1. However, as described in the introduction, it is highly unlikely to observe such extensive movements within the timescales reachable with cMD simulations. We thus apply a simulation protocol where enhanced and classic sampling techniques are combined to model the conformational rearrangements along the transition pathway to the locally unfolded conformation.

We visualize the captured free energy surfaces in a combined PCA space (see Methods). The extend of this surface thus reflects all captured structural variances and allows a direct comparison. We find that within this projection, *Bet v 1a-D69I* clearly displays the most restricted conformational space. *Bet v 1a* and *Bet v 1d* cover areas of similar extent, but the free energy surface of *Bet v 1d* presents itself as generally more favorable than *Bet v 1a*. This indicates that while similar conformational states are accessible for *Bet v 1a* and *Bet v 1d*, they are much more likely to occur in the ensemble of *Bet v 1d*. These qualitative observations can be quantified via transition timescales and stationary distributions from the MSM. The decline of transition timescales from *Bet v 1a-D69I* (57 μs), over *Bet v 1a* (21 μs) to *Bet v 1d* (9 μs) shown in Figure 3B directly corresponds to a decrease in barrier height between the folded and partially unfolded conformational states. However, the essential information of the constructed model lies in the thermodynamics, i.e., the probabilities, of the dynamic equilibrium between the native fold and the partially unfolded conformational states. Consistent with the previously postulated hypothesis, we find the highest population for the folded state in *Bet v 1a-D69I*, the stabilized variant, with 95 ± 2%. The wild-type protein *Bet v 1a* shows a slightly lower probability of 87 ± 3% for the folded state. The proteolytically least stable variant, *Bet v 1d*, indeed exhibits by far the lowest probability of being in the fully folded state with 65 ± 6%. These findings support the long-standing postulation of a link between proteolytic susceptibility and protein dynamics, i.e., local unfolding events (Freier et al., 2015; Nakamura et al., 2016; Scheiblhofer et al., 2017).

Based on sequence or a single structure alone, it is challenging to comprehend how a few mutations far from the cleavage site result in a substantial shift in proteolytic cleavage rates. However, in describing the Bet v 1 variants as conformational ensembles we find a coherent link between local unfolding probability and proteolytic susceptibility. In many experimental and computational studies this relation has already been debated (Parsell and Sauer, 1989; Thai et al., 2004; Ohkuri et al., 2010; Asam et al., 2014; Grutsch et al., 2014, 2017; Scheiblhofer et al., 2017). Yet, to the best of our knowledge, to this point no structural model of a partially unfolded state has been suggested. Here, we demonstrate that with the current advances simulation techniques and computational resources it is now possible to sample extensive portions of a proteins conformational space, including partial unfolding.

The presented workflow builds upon specific experimental data. On the one hand, a reliable starting structure for the protein in focus is imperative to perform MD simulations in general. On the other hand, this particular approach relies on knowledge of early cleavage sites, which are determinant for the initial processing of the allergen and in consequence are the focus of this study. However, in principle the presented approach is generalizable and broadly applicable. Furthermore, the required data on structure and proteolytic stability is already available for a broad range of allergen proteins (Gadermaier et al., 2011; Toda et al., 2011; Kitzmuller et al., 2015; Pablos et al., 2018). We consider the presented study as a proof of concept, that once this data is available, it is possible estimate how a particular mutation, no matter how far away from the cleavage site, impacts the cleavage rate. We thus envisage an iterative workflow where initial experimental data on the structure and proteolytic stability of one protein builds the foundation for computational models on the proteolytic stability of a multitude of mutants. These predictions can then be used to guide the experimental efforts toward the most promising candidates. The resulting cleavage data in turn can be used to refine the models and enhance prediction accuracy. Similar strategies have already demonstrated the reliability of predictions from MD simulations combined with MSM analysis on the impact of point mutations on protein stability (Zimmerman et al., 2018). Given the tremendous clinical relevance of allergen proteins we thus suggest physics-based computational models as promising tools for the design recombinant proteins for allergen-specific immunotherapy (Curin et al., 2018).

## Conclusions

In this study, we employ classic and enhanced sampling techniques to test whether we are able to characterize the relation between protein dynamics and proteolytic susceptibility. Mimicking the environmental conditions of endolysosomal degradation, we observe a link between the probability of partial unfolding and proteolytic susceptibility for three Bet v 1 variants.

Based on experimental data we estimate how a particular mutation, no matter how far away from the cleavage site, impacts the conformational ensemble and thus cleavage rate. Further testing will be necessary to optimize and strengthen the presented protocol; however, the required experimental data are already available for a broad range of allergen proteins and the approach is fully generalizable. The relation of proteolytic susceptibility and immunogenicity of exogenous proteins presents a promising opportunity for the design of recombinant proteins as vaccines for allergen-specific immunotherapy (Marth et al., 2014; Valenta et al., 2016; Scheiblhofer et al., 2017). With this study, we demonstrate the feasibility of employing MD simulations to characterize structural ensembles of allergen proteins in the context of proteolytic stability.

## Data Availability Statement

All datasets generated for this study are included in the article/Supplementary Material.

## Author Contributions

All authors listed have made a substantial, direct and intellectual contribution to the work, and approved it for publication.

### Conflict of Interest

The authors declare that the research was conducted in the absence of any commercial or financial relationships that could be construed as a potential conflict of interest.

## Abbreviations

Bet v 1: major birch pollen allergen (betula verrucosa)
MD: molecular dynamics
MHC2: major histocompatibility complex molecules type 2
NMR: nuclear magnetic resonance
cMD: classic molecular dynamics
MDMD: metadynamics molecular dynamics
PDB: protein data bank
MSM: Markov state model
PCA: principle component analysis
tICA: time-lagged independent component analysis
PCCA+: Perron cluster cluster analysis
RMSD: root mean squared distance
RMSF: root mean squared fluctuation
