# Supplementary material for: Dynamics Rationalize Proteolytic Susceptibility of the Major Birch Pollen Allergen Bet v 1
Source: Front Mol Biosci. 2020 Feb 20;7:18. doi: 10.3389/fmolb.2020.00018 (PMC7045072; doi:10.3389/fmolb.2020.00018)
Supplement: Supplementary file 1 [file Data_Sheet_1.docx]

Supplementary Material


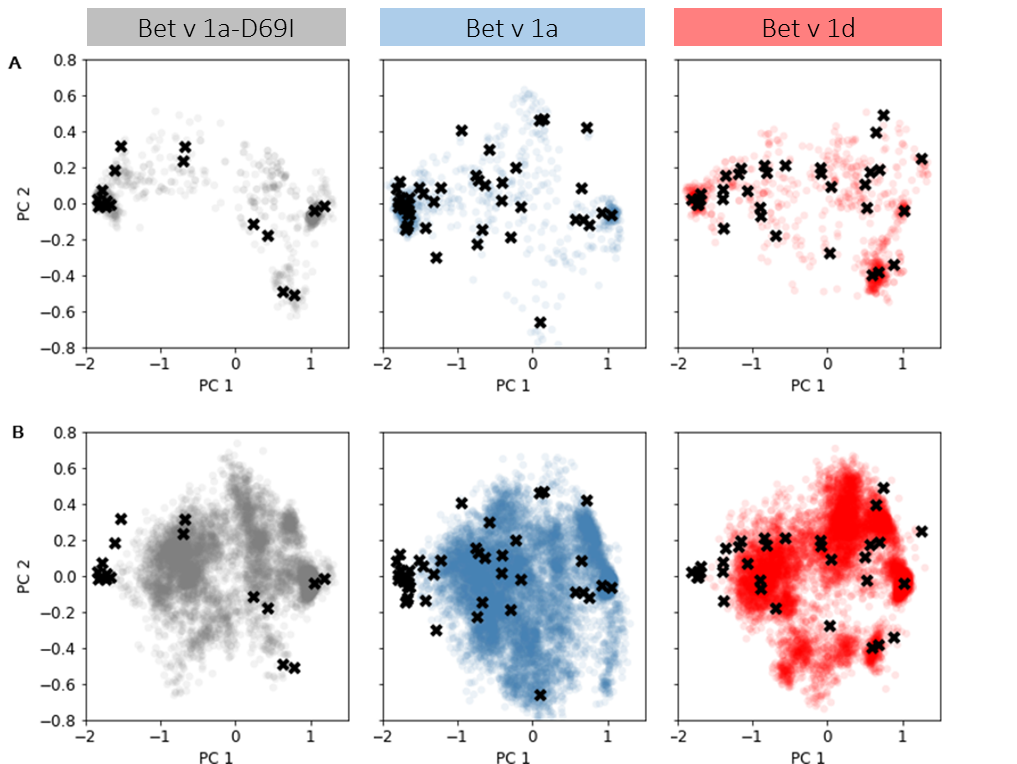


**Supplementary Figure 1.** **(A)** Conformational ensembles captured during well-tempered MDMD simulations are projected onto the combined PCA space and depicted in color. Black crosses mark representative structures, which were used to seed classic MD simulations. **(B)** Structural information resulting from seeded classic MD simulations projected onto the combined PCA space and depicted in color.


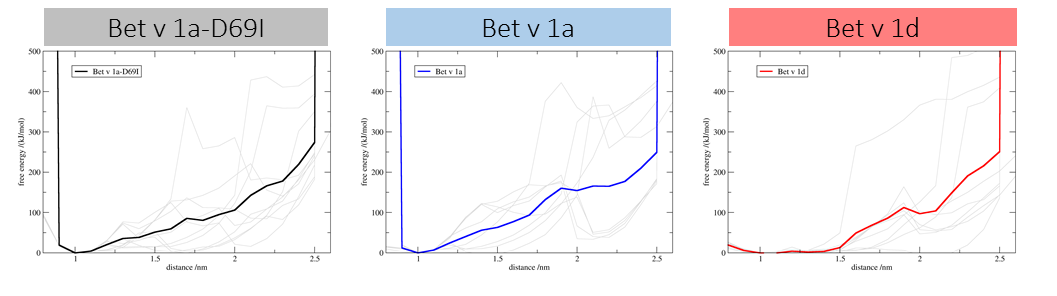
**Supplementary Figure 2.** Reweighted free energy of distance used as CV in MDMD simulations. For each system the individual curve of each run is depicted in gray and their average is shown in color.


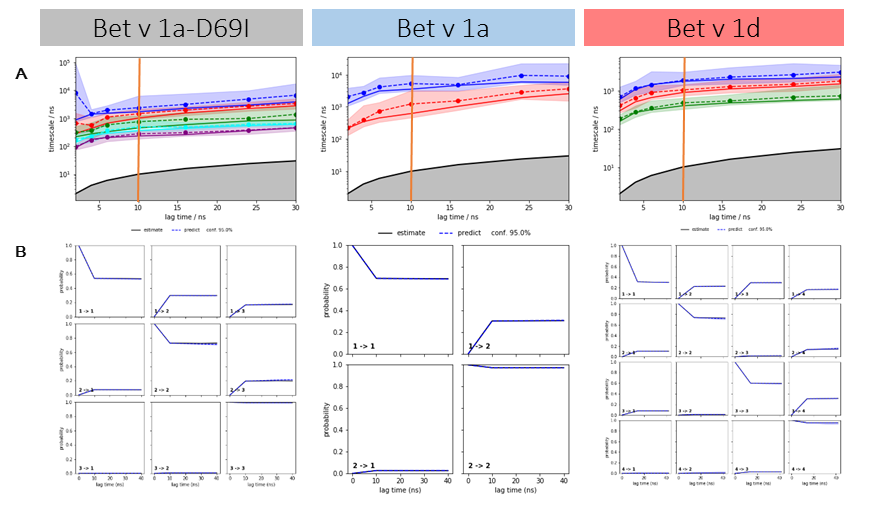


**Supplementary Figure 3.** MSM validation tools. **(A)** Implied timescales as function of lag time with the selected lag time of 10 ns highlighted by the orange vertical line. **(B)** Chapman-Kolmogorov tests for the respective MSM of each system.


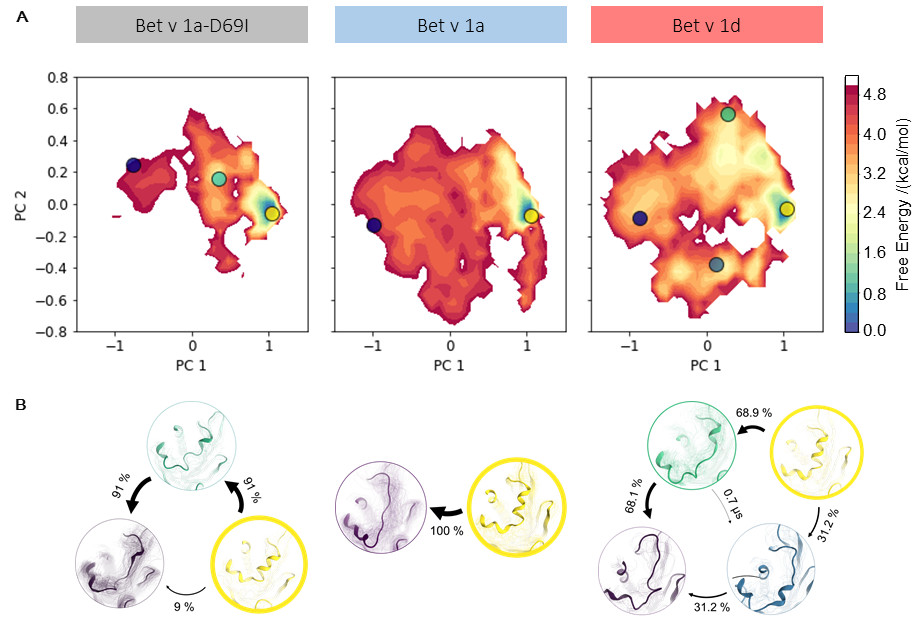


**Supplementary Figure 4.** **(A**) Free energy landscape during local unfolding. The accumulated structural information from the seeded cMD simulations were color-coded according to the reweighted free energies and projected onto the first two eigenvectors of the combined PCA space. **(B)** The pathway of partial unfolding is depicted according to the net reactive flux calculated from the Bayesian MSM.

Supplementary Table 1 Mean first passage times including respective standard deviations calculated from the Bayesian MSM for each system. Folded and unfolded states are color coded according to Figure 3.

| Bet v 1a-D69I | | | | | |
| --- | --- | --- | --- | --- | --- |
|  | Folded | Unfolded 0 | Unfolded 1 |  | |
| Folded |  | 530.6 ± 138.2 µs | 56.5 ± 25.6 µs |  | |
| Unfolded 0 | 3.7 ± 0.9 µs |  | 6.0 ± 3.1 µs |  | |
| Unfolded 1 | 1.9 ± 0.6 µs | 468.7 ± 122.1 µs |  |  | |
| Bet v 1a | | | | | |
|  | Folded | Unfolded 0 |  | |  |
| Folded |  | 20.9 ± 7.3µs |  | |  |
| Unfolded 0 | 2.5 ± 1.2 µs |  |  | |  |
| Bet v 1d | | | | | |
|  | Folded | Unfolded 0 | Unfolded 1 | Unfolded 2 | |
| Folded |  | 40.5 ± 10.9 µs | 40.3 ± 23.7 µs | 9.1 ± 4.2 µs | |
| Unfolded 0 | 4.9 ± 0.8 µs |  | 23.1 ± 12.7 µs | 2.7 ± 1.0 µs | |
| Unfolded 1 | 4.2 ± 1.5 µs | 22.1 ± 6.9 µs |  | 7.9 ± 3.0 µs | |
| Unfolded 2 | 2.6 ± 0.6 µs | 29.3 ± 9.6 µs | 38.3 ± 21.4 µs |  | |

Supplementary Table 2 Probability and standard deviations of the metastable states calculated from the Bayesian MSM of each system. Folded and unfolded states are color coded according to Figure 3.

| Bet v 1a-D69I | |
| --- | --- |
| Folded | 95 ± 2 % |
| Unfolded 0 | 0.5 ± 0.2 % |
| Unfolded 1 | 4.1 ± 2.1 % |
| Bet v 1a | |
| Folded | 87 ± 3 % |
| Unfolded 0 | 13 ± 3 % |
| Bet v 1d | |
| Folded | 65 ± 6 % |
| Unfolded 0 | 3.4 ± 0.7 % |
| Unfolded 1 | 5.3 ± 0.2 % |
| Unfolded 2 | 26 ± 6 % |


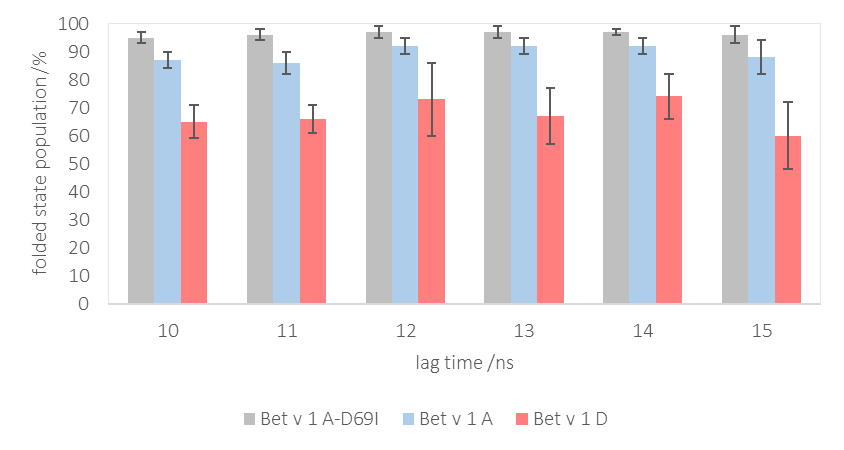


**Supplementary Figure 5.** Population of the folded state at varying lag times, error bars represent standard deviations calculated from the Bayesian MSM of each system at the respective lag time.
